# Somatosensory false feedback biases emotional ratings through interoceptive embodiment

**DOI:** 10.1038/s41598-025-94971-6

**Published:** 2025-04-03

**Authors:** Joel Patchitt, Sarah Garfinkel, William H. Strawson, Mark Miller, Manos Tsakiris, Andy Clark, Hugo D. Critchley

**Affiliations:** 1 Sussex Centre for Consciousness Science, University of Sussex, Brighton, BN1 9QJ UK; 2 Department of Clinical Neuroscience, Brighton and Sussex Medical School, University of Sussex, Brighton, BN1 9RY UK; 3 Institute of Cognitive Neuroscience, University College London, London, WC1N 3AZ UK; 4 Monash Centre for Consciousness and Contemplative Studies, Monash University, Melbourne, VIC 3168 Australia; 5 Psychology Department, University of Toronto, Toronto, ON M5S 2E5 Canada; 6 Department of Psychology, Royal Holloway, University of London, Egham, TW20 0EX UK; 7 School of Media, Arts and Humanities, University of Sussex, Brighton, BN1 9RG UK; 8 School of Engineering and Informatics, University of Sussex, Brighton, BN1 9QG UK

**Keywords:** Interoception, False physiological feedback, Insula, Emotion recognition, Perception, Predictive coding, Biotechnology, Neuroscience, Physiology, Psychology

## Abstract

Mismatches between perceived and veridical physiological signals during false feedback (FFB) can bias emotional judgements. Paradigms using auditory FFB suggest perceived changes in heart rate (HR) increase ratings of emotional intensity irrespective of feedback type (increased or decreased HR), implicating right anterior insula as a mismatch comparator between exteroceptive and interoceptive information. However, few paradigms have examined effects of somatosensory FFB. Participants rated the emotional intensity of randomized facial expressions while they received 20 s blocks of pulsatile somatosensory stimulation at rates higher than HR, lower than HR, equivalent to HR, or no stimulation during a functional magnetic resonance neuroimaging scan. FFB exerted a bidirectional effect on reported intensity ratings of the emotional faces, increasing over the course of each 20 s stimulation block. Neuroimaging showed FFB engaging regions indicative of affective touch processing, embodiment, and reflex suppression. Contrasting higher vs. lower HR FFB revealed engagement of right insula and centres supporting socio-emotional processing. Results indicate that exposure to pulsatile somatosensory stimulation can influence emotional judgements though its progressive embodiment as a perceived interoceptive arousal state, biasing how affective salience is ascribed to external stimuli. Results are consistent with multimodal integration of priors and prediction-error signalling in shaping perceptual judgments.

**Supplementary Information:**

The online version contains supplementary material available at 10.1038/s41598-025-94971-6.

## Introduction

Subjective emotional experience is proposed to arise from integrating information about bodily state (e.g. changes in physiological arousal) with contextual information from the external environment that might have evoked such bodily changes, e.g. by virtue of being threatening, appetitive or safe. Conversely, changes in bodily arousal can evoke physiological feelings that imbue, through association, affective meaning to other stimuli. This influential model of emotion^1–4^, thereby qualifies more reductionist physiological accounts^5,6^. Empirical evidence of (first level) physiological effects on emotional phenomenology includes observed increases in self-reported level of sexual arousal when viewing erotic material after physical exercise^7,8^. However, second-level conscious appraisal of changes in bodily arousal can generate more nuanced emotional experience. For example, pharmacologically induced cardiorespiratory arousal may be differentially interpreted as anger or elation depending on the wider social context^4^.

False physiological feedback (FFB) can influence perceptual judgement of emotional material. The conscious representation of (mis)information about physiological signals arguably ‘overrides’ veridical interoceptive information. In a historical illustration, male participants rated pictures of naked women as more attractive when played auditory signals that suggested bi-directional changes in physiological arousal^9^. Such effects do not rely on physiological entrainment but reflect higher order conscious appraisal of information about one’s own physiological state^10–13^. FFB arguably makes stimuli more salient via a mismatch with veridical afferent signals^14,15^, in which ‘unaccounted arousal’ may enhance attention to visceral and external evidence^12,16,17^ through generation of interoceptive prediction errors^18^.

Anterior insula, particularly in the right hemisphere (RAI) is implicated as the cortical substrate for representing and integrating interoceptive signals for second-level representation as conscious feelings states, including emotional experiences^19–22^. Primate studies, mapping spinothalamocortical and ascending brainstem interoceptive inputs into insular cortex provide anatomical endorsement of this functional attribution^13,19,23^. Activity in the RAI is associated with interoceptive sensitivity in heartbeat detection tasks^21,24^ including attention-dependent processing of exteroceptive/interoceptive mismatches during asynchronous feedback^21^. In this context, unattributed or unpredicted states of arousal may engender anxiety states through insular engagement^18^, often in conjunction with ‘visceromotor’ anterior midcingulate cortex^25^. Asynchronous auditory FFB of heartbeats, delivered faster or slower than actual heartrate, was observed to amplify emotional intensity ratings of neutral faces (over strongly happy or angry faces)^11^. This effect was predicted by the degree of activation with RAI and amygdala, suggesting second-level neural interpretations of physiological arousal help resolve affective ambiguity.

Published FFB paradigms often use an external auditory signal to induce a mismatch between veridical and perceived heart rate^9,11,21^. Of the very few paradigms that have examined effects of somatosensory stimulation within the context of FFB on emotional outcomes, a simulated decrease in cardiovascular arousal state was observed to attenuate the experience of anxiety within social situations^26^: Delivery of pulsating vibrotactile stimulation (at a lower than resting HR) to the wrist during a socially stressful challenge led participants to report lower levels of anxiety, compared to controls who received no stimulation. Electrodermal measures of physiological arousal were attenuated yet there were no differences in HR between active and control conditions. This finding reinforced the view that appraisal of physiological/external information, rather than physiological entrainment, was responsible for emotion FFB effects, regardless of modality. Unlike studies using auditory FFB, effects of somatosensory-driven FFB were directional, ameliorating anxiety when FFB was presented at lower than veridical HR. This effect suggests that somatosensory feedback (compared to auditory FFB) is proximally easier to integrate and interpret within interoceptive representations of physiological arousal. Somatosensory information is typically much closer in its contribution to physiological regulation and representation of the body, directly giving rise to feeling states (including contributions to heartbeat sensations). As such, we predicted a stronger more meaningful effect of somatosensory mediated false physiological feedback on affective processes.

The present study examines how the effects of somatosensory FFB can modulate the appraisal of social affective information (emotional judgement of facial expressions), including both behavioural outcomes and neural correlates. We used an fMRI-compatible device to deliver pulsing vibro-tactile (heartbeat-like) FFB to simulate increased and decreased heart rate (i.e. states of cardiovascular arousal) during a task in which participants rated their perception of the emotional intensity of different face pictures. The stimuli depicted positive (happy), and negative (angry, fearful) faces graded across an expressive range through morphing of neutral faces. Concurrently, we used functional magnetic resonance imaging (fMRI) to probe the central neural mechanisms associated with FFB and relate them to the behavioural outcomes of the paradigm.

We hypothesized that; (1) heartbeat-like somatosensory FFB will exert bi-directional effects on emotional intensity decisions depending on whether FFB frequency is delivered higher or lower than veridical HR; (2) this effect will occur independent of HR entrainment to FFB; (3) RAI (as a hub integrating veridical and falsified physiological information) will be engaged in the representation of FFB and its impact on emotional judgments.

## Methodology

###  Participants

We recruited 41 non-clinical adult volunteers (n females = 29) between 18 and 65 years old (M = 29.7, SD = 15.8). Two participants were excluded from the BOLD analyses (*n* = 39): One missing behavioural data (excluding them from the behavioural analyses (*n* = 40)) and the other a corrupted BOLD acquisition. For full demographics and inclusion/exclusion criteria, see supplementary Materials. This study was approved by the Research Governance and Ethics Committee at the University of Sussex and was performed in accordance with relevant named guidelines and regulations.

### Stimuli

**Fig. 1:**
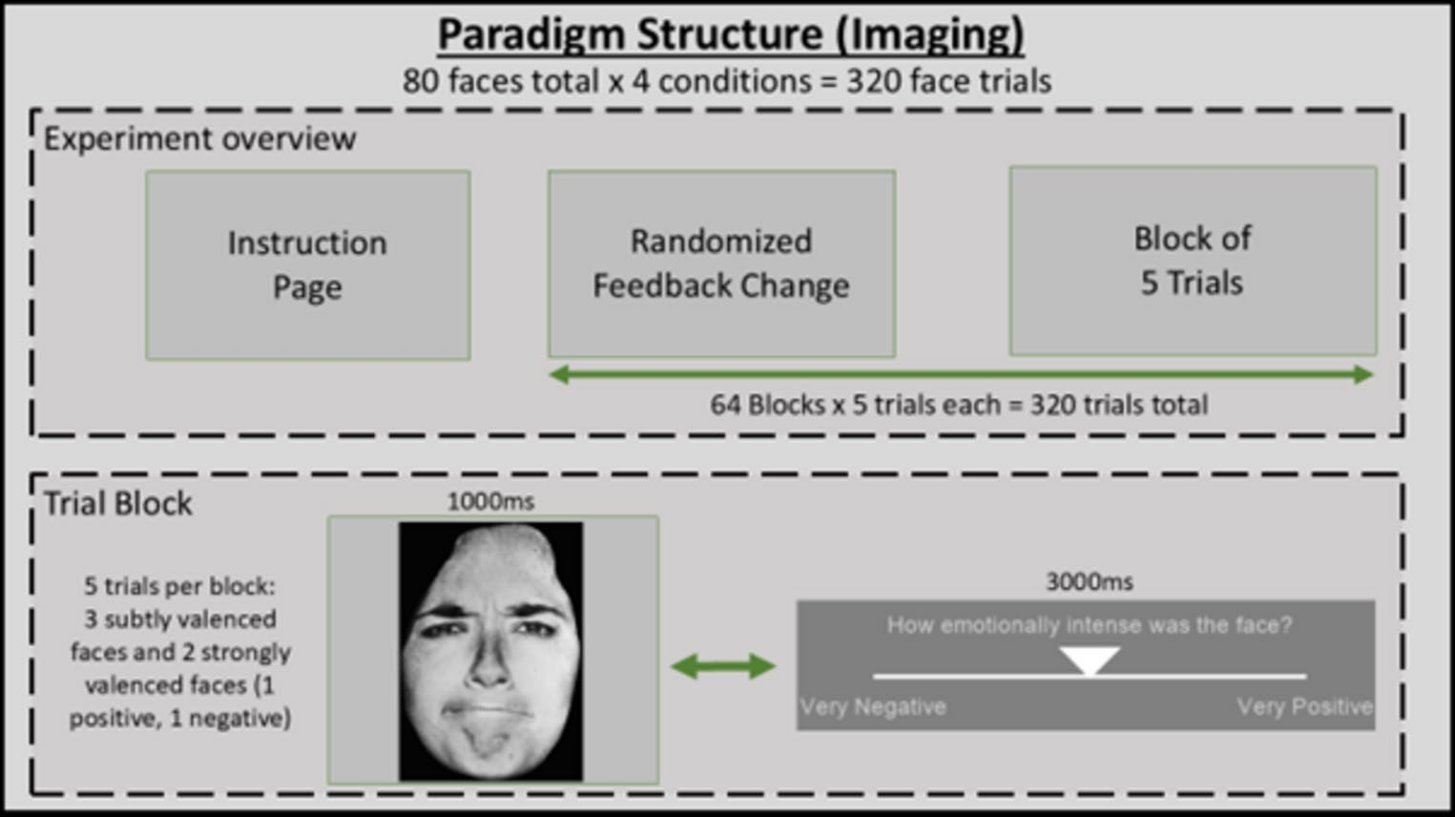
Visualisation of fMRI BOLD task. Face stimuli (1000ms) and rating screen (3000ms) alternate between each other with Feedback condition changing every 5 iterations for a total of 64 blocks. Each block was pseudorandomised to ensure subjects were exposed to 3 expressions very close to neutral but slightly positively/negatively valanced (labelled neutral) and 2 strongly valenced expressions (labelled positive/negative).

Eighty face images were selected from The Eckman and Friesen Pictures of Facial Affect emotional face database^27^ comprising four individual identities, two of each gender, ranging from no emotional valence (‘neutral’) to strongly valenced facial expressions. Images were cropped and processed, to derive a graded range of facial expressions both happy (10 per person) and frightened or angry faces (10 per person), that was biased towards more subtle (ambiguous) expressions (see Supplementary Material). The majority of these faces depicted expressions that were only slightly positively/negatively valenced, with a portion of strongly valenced expressions mixed into each stimulation block (labelled positive/negative in Fig. 1), introducing significant ambiguity into each block^7^. It should be noted that the faces presented in this paradigm represented emotional valence on a continuum of intensity ranging from emotionally ambiguous (neutral) to strongly valanced. False feedback was presented using a fMRI safe vibration device that presented pulsatile sensations at a periliminal intensity. This was achieved by starting at an intensity below conscious perception and slowly increased to the degree that the participant could only slightly attend to the vibration. Once participants moved their attention away from the vibration it could no longer be felt.

### Procedure

Each participant underwent a pre-screening phone call (after reading the participant information sheet) in which strict eligibility for the neuroimaging arm of the study was assessed. After which, an invitation was extended to visit the Clinical Imaging Sciences Centre (University of Sussex) where the participant was led through the informed consent procedure and completed a series of safety checks. Before entering the scanner, the participant re-read the participant information sheet, asked any questions, and signed the imaging consent form. A 3-lead electrocardiogram (ECG) was then fitted to the participant’s chest in an L shape around the left breast, alongside a pulse oximeter on the left hand and false feedback device on the right. The response button box was also placed in the right hand of the participant.

First, 12 minutes of task-free (‘resting-state’) T2*-weighed BOLD imaging was acquired (analysed elsewhere) followed by 1 minute of spin echo field maps for susceptibility distortion correction. After which the 23 minute fMRI BOLD data was acquired during the FFB task.

The FFB task required participants to perform simple emotional intensity rating judgments of face pictures that were presented on a screen made visible through the scanner bore. Eighty faces of varying gender (male/female) and emotion (happy/fearful/angry) were shown in blocks of five, with FFB condition changing at the end of each block. Each face was presented for 1000ms immediately switching to a visual analogue scale (VAS) rating screen for 3000ms (see Fig. 1) repeating until all five faces in the block were shown. The VAS scale gave participants the option to rate each face positively in degrees of intensity (1, 2, 3, 4) or negatively in degrees of intensity (-1, -2, -3, -4) by pressing right and left buttons respectively on a button box placed in their right hand. Alternatively, no button press would indicate a neutral face. The same 80 faces were used in all four conditions.

Conditions included false physiological feedback at a static BPM representing (1) 20% faster than participant’s veridical HR (HIGHER) (2) 20% slower than participant’s veridical HR (LOWER) (3) static representation of the last 20 s of the participant’s measured HR (SAME) (4) no physiological feedback (NULL). These four conditions were randomly cycled throughout the experiment 16 times each, changing every 20 s (5 trials x 4 s) for a total of 64 blocks, resulting in 320 subjective intensity ratings per participant. The FFB did not emulate the variable inter-beat interval (IBI) normally present during natural heart pulsation. The above capitalised terms are from this point forward only used in their capitalised form when speaking specifically about the defined condition it has been linked to.

After the fMRI BOLD sequence, a 7-minute NODDI diffusion-weighted imaging sequence was acquired (analysed elsewhere), followed by a T1w and T2w Structural image sequences. Once the participant had left the scanner, they were debriefed and received payment for participation.

### fMRI data acquisition and analyses

#### Scanning parameters

Neuroimaging data was collected using a 3T Siemens scanner at the Clinical Imaging sciences Centre based in the University of Sussex. For a full list of scanning parameters see Supplementary Materials.

#### Pre-processing of neuroimaging data

BOLD EPI datasets were pre-processed initially using f*MRIPrep*21.0.0 [RRID: SCR_016216], which is based on *Nipype*1.6.1 [RRID: SCR_002502]^28^ and subsequently finalised using FSL^29,30^ (See Supplementary Materials).

#### Neuroimaging analytic design and analysis

During the fMRI analysis we were interested in (a) The general effect of somatosensory false feedback in the brain, (b) How this effect interacts with the perception of valence, (c) Whether any of these regions are parametrically modulated by exposure to somatosensory FFB over time. For this reason, the general linear model initially followed a non-valenced blocked design consisting of fixed length, stochastically presented physiological ‘Feedback’ explanatory variables (EVs) (01: HIGHER, 02: LOWER, 03: SAME, 04: NULL) (Feedback Model). We then increased the granularity in a separate model by including emotional valence (01: POSITIVE, 02: NEGATIVE), resulting in 8 EVs (Valence Model). These EVs were parametrically modulated by Trial resulting in a separate set of EVs (Exposure Model) for the exposure related analysis by mean-centring the ‘Trial’ (1 to 5) in which the event occurred. This was to account for exposure effects seen in the behavioural data (see Sect. Effect of exposure to false physiological feedback on subjective intensity ratings over time.; Fig. 3). Each EV was convolved with a double gamma function to model the hemodynamic responses.

Functional neuroimaging data analysis was conducted using FSL FEAT^29,30^. General linear modelling (GLM) was conducted to ascertain which voxels’ blood oxygenation level–dependent (BOLD) signal was associated with the EV’s of interest. The first level design matrices constructed for each participant consisted of a canonical gamma hemodynamic response function (FSL) for each contrast (see RESULTS Sect. 3.3.) To reduce the error variance produced by hemodynamic timing, temporal derivatives were included in the design and were subsequently left out of the second level analysis.

Subsequent second-level analyses utilised a mixed effects design to conduct a higher-level group analysis on the first level contrasts. Group level maps employed a cluster forming threshold of Z > 2.3 (*P* < .01) and an alpha value of *P* < .05 to correct for comparisons at a cluster-wise level. Runs from all 39 participants were included in the higher-level analysis.

## Results

### Behavioural results

#### Effects of false physiological feedback (FFB) on ratings of perceived emotion

**Fig. 2:**
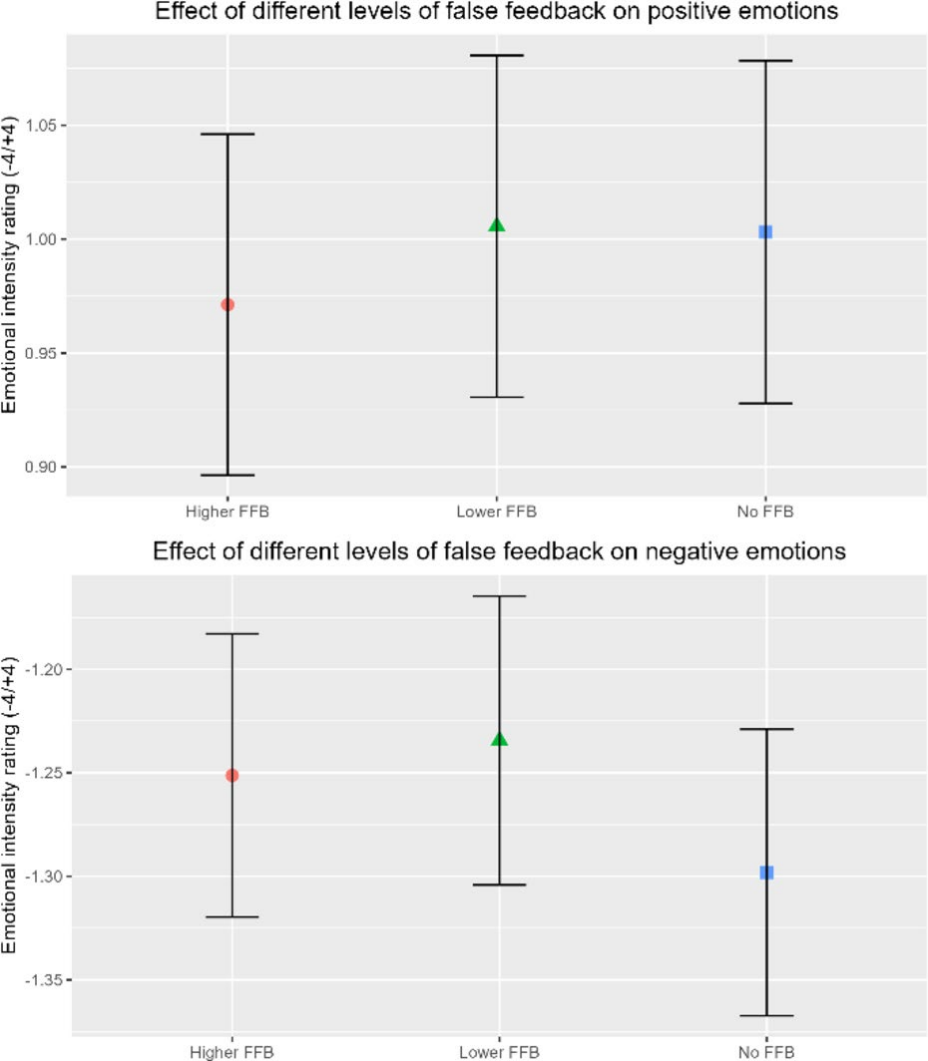
Mean Effect of false physiological feedback on subjective ratings of intensity with 95% confidence interval error bars. Stimuli displaying a positive valence face are rated significantly more positive than stimuli displaying a negative valence face.

We first tested for effects of FFB, on emotional intensity ratings of face stimuli using a 3 × 2 ANOVA using Condition (HIGHER, LOWER, NULL) and Emotion (POSITIVE, NEGATIVE) as explanatory variables. Assumptions for normality, homogeneity of variance and independence was met after inspection of the histograms, Q-Q plots and box plots and a random sampling design was used. We confirmed a main effect of Emotion *F*(1, 39) = 313, *p* < .001, ηp2 = 0.885, such that POSITIVE faces were rated more positive than NEGATIVE faces (Mdiff +/- SEdiff = 2.255 +- 0.019). We observed no average effect of Condition *F*(1.64, 63.84) = 1.309, *p* = .274, ηp2 = 0.027 nor interaction between Condition and Emotion *F*(1.67, 64.96) = 2.304, *p* = .113, ηp2 = 0.050. These data suggest that there was no average impact of FFB delivered in 20s blocks, on the ratings of the emotional intensity of facial expressions (see Fig. 2). These initial absent/subthreshold effects of feedback conditions motivated more granular analysis of time-dependent effects described below.

#### Effect of exposure to false physiological feedback on subjective intensity ratings over time

Following the concept of repetition suppression and model formation, asserted by the predictive coding framework^31,32^, we proposed that any effect of FFB is likely to develop over time as the repeated processing of stimuli updates one’s own interoceptive model. Visualisation of our data indicated an effect of FFB exposure over time on intensity ratings, i.e. the impact of FFB emerged over the course of the 20s stimulation blocks. Due to the nature of trial being a linearly spaced ordinal variable, we opted to formally test this hypothesis using a linear mixed model analysis of variance conducted with two fixed effect categorical factors: Condition (HIGHER, LOWER, SAME, NULL) and Emotion (POSITIVE, NEGATIVE) and one fixed effect continuous factor: Trial number (1, 2, 3, 4, 5) (reflecting stimulus position in time over each 20 FFB blocks). ‘Participant’ was included as a random effects factor. The assumptions of linearity, sphericity, homoscedasticity, normality of residuals, random effects normality, homogeneity of variance and multi-collinearity were met through inspection of residual vs. fitted plots, residuals vs. predictor plots, Q-Q plots of residuals and random effects, histogram of residuals and VIF values for all predictor combinations below 5. We found evidence for a significant interaction between Condition, Emotion and Trial *F*(3) = 47.865, *p* < .001.

**Fig. 3:**
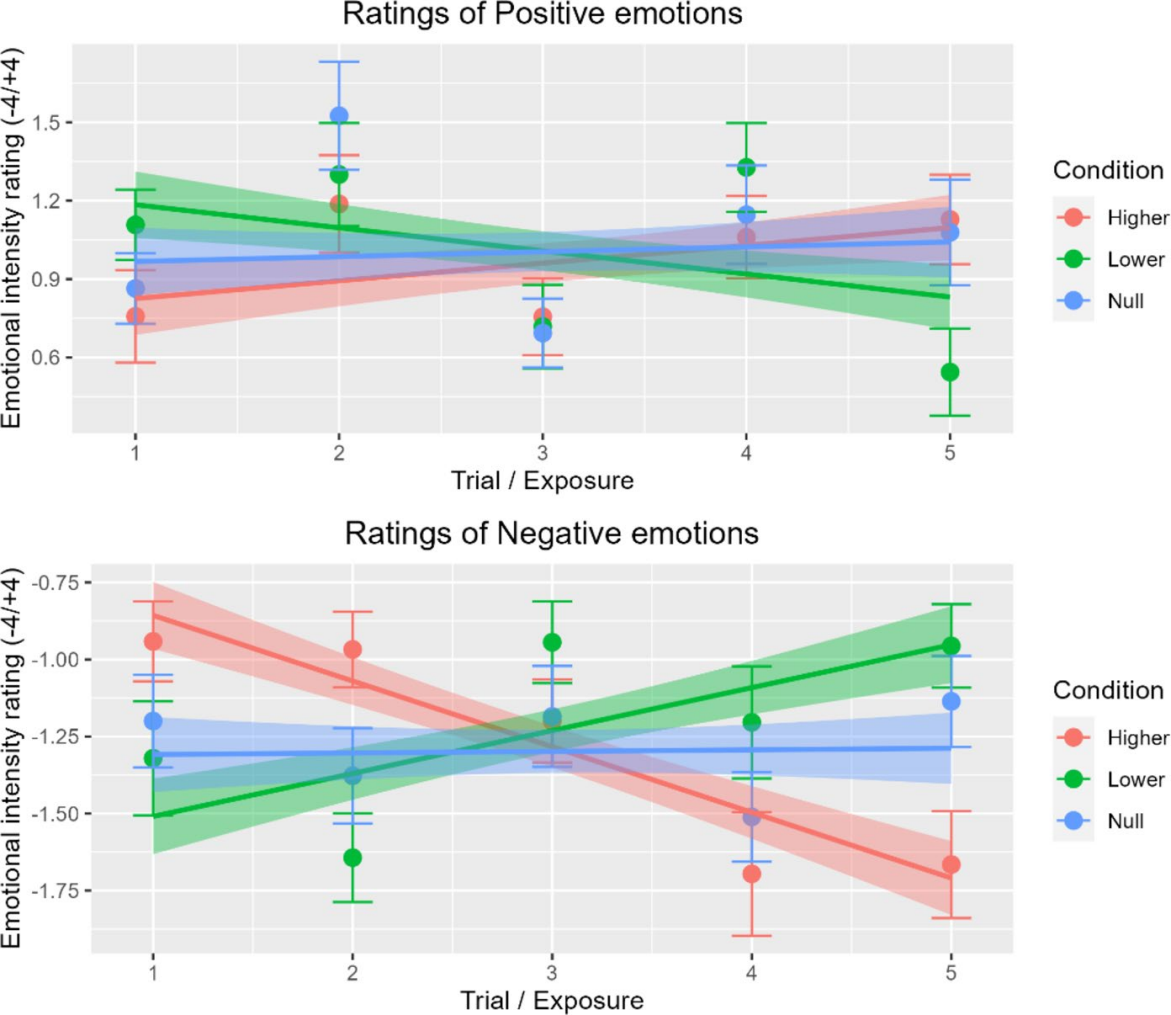
Bi-directional exposure effect of tactile false physiological feedback on mean subjective intensity ratings of emotionally ambiguous faces over time with 95% confidence interval error bars. HIGHER FFB exerts increases in mean intensity ratings over time for both positive and negative emotions. LOWER FFB exerts the opposite effect.

To understand this interaction further, we ran three Bonferroni-corrected post hoc linear mixed models analyses of variance, split by the four levels of Condition. These revealed significant interactions between Emotion and Trial in the HIGHER FFB, LOWER FFB and SAME FFB conditions but not for the NULL FFB condition (see Table 1; Fig. 3). Our initial approach would be to interpret the effects of HIGHER and LOWER conditions in contrast to SAME condition as a baseline. Unfortunately, the SAME FFB condition was not synchronous to veridical HR but rather presented at a static BPM. Many studies have linked asynchronous vs. synchronous heart rate feedback to differences in emotional decision making^33–35^, and while HIGHER and LOWER conditions likely share a similar spread of synchronous vs. asynchronous beat occurrences, they represent a distinct change in the participants arousal, exerting bi-directional changes in emotion perception through mismatch in overall rate and beat synchrony. Nevertheless, FFB presented at the participants HR encompasses mismatch in beat synchrony, which limits the degree to which the SAME FFB condition can be used as a sanitary baseline for addressing the hypotheses. For clarity, we chose to remove the same condition from Fig. 3 (See Supplementary Material for Fig. 3 with SAME FFB). Thus, once time-dependent effects were considered, FFB at rates faster and slower than HR were shown to bias the rating of emotional faces. This effect was also replicated in an alternative analysis approach using least-squares means (See supplementary material).

**Table 1:** Post hoc LMM analyses of variance for Condition*Emotion*Trial interaction on subjective intensity ratings.

	F(1)	*p*
HIGHER False Physiological Feedback	62.185	< 0.001
LOWER False Physiological Feedback	39.375	< 0.001
SAME False Physiological Feedback	49.227	< 0.001
NULL No False Physiological Feedback	0.14	0.708

Six simple linear regressions were conducted, correcting for multiple comparisons using the Bonferroni method, with Trial as the predictor for HIGHER, LOWER and SAME conditions, split by Emotion. These revealed that FFB representing an increased arousal state (relative to veridical HR) enhanced subjective emotional intensity ratings for NEGATIVE stimuli but not for POSITIVE stimuli. Conversely, FFB representing a reduced arousal state evoked a decrease in subjective emotional intensity ratings in both POSITIVE and NEGATIVE stimuli (see Table 2). SAME FFB elicited a decrease in intensity ratings in POSITIVE stimuli, but this is likely to be sporadic due to the static BPM presentation^33–35^.

**Table 2:** Bonferroni corrected simple linear regressions of trial split by condition and emotional Valence on subjective intensity ratings.

	R2	F(1, 1598)	β	*p*	95% lower	95% upper
HIGHER POSITIVE	0.004	5.95	0.068	0.015	0.013	0.122
HIGHER NEGATIVE	0.049	81.53	−0.213	< 0.001	−0.259	−0.167
LOWER POSITIVE	0.007	11.66	-0.088	< 0.001	-0.139	−0.038
LOWER NEGATIVE	0.018	28.5	0.139	< 0.001	0.088	0.190
SAME POSITIVE	0.041	68.47	−0.219	< 0.001	−0.272	−0.168
SAME NEGATIVE	0.001	1.93	0.036	0.16	−0.015	0.087

These data indicate that the effects of exposure to FFB on subjective intensity ratings of valence is greater for negative valence stimuli than they are for positive valence stimuli. Additionally, for positive valence stimuli, FFB has more of an effect at reducing positive subjective ratings than increasing them.

### Physiological entrainment

**Fig. 4:**
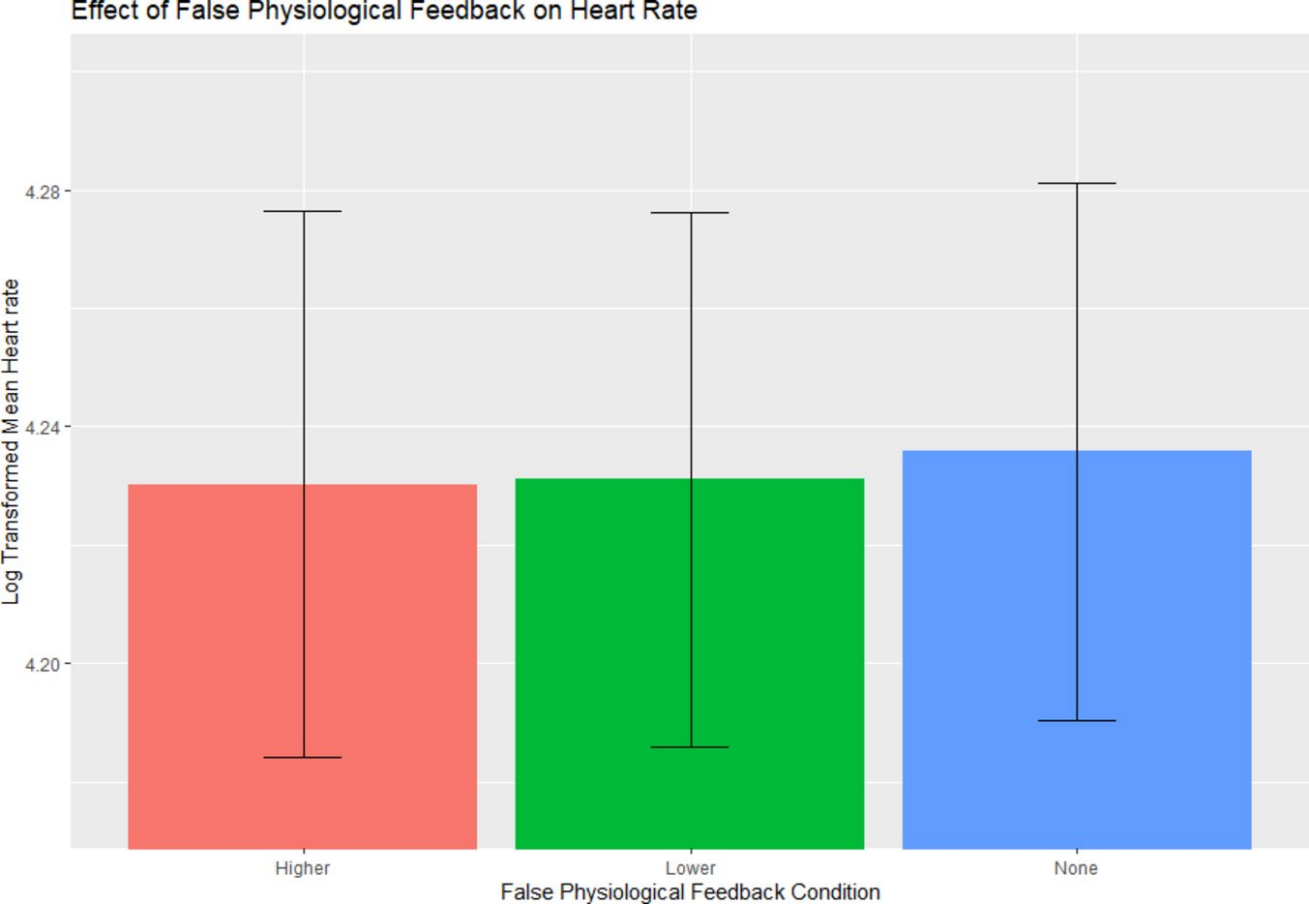
Mean effect of false physiological feedback conditions on heart rate with 95% confidence interval error bars. Heart rate during HIGHER and LOWER FFB blocks are significantly lower than during no feedback blocks.

To test for any entrainment of the heart to the FFB we ran a repeated measures one way ANOVA with log transformed heart rate as the dependant variable and condition (HIGHER, LOWER, NULL) as the explanatory variable. We found a significant main effect of condition *F*(2, 58) = 5.594, *p* = .007. Post hoc Bonferroni correction t-tests revealed that both HIGHER (*p* = .028) and LOWER (*p* = .023) conditions were significantly different from the NULL FFB, but not significantly different from each other (*p* > .99) such that heart rate during HIGHER and LOWER FFB conditions was slower compared to NULL FFB (see Fig. 4). This suggests that any effects of FFB on heart rate were not entrainment-related and were unlikely to have contributed to the behavioural results.

### Neural correlates of false physiological feedback (FFB)

#### Posterior insular, secondary somatosensory, premotor and sensorimotor cortical activation associated with somatosensory stimulation emulating heart rate feedback

**Fig. 5:**
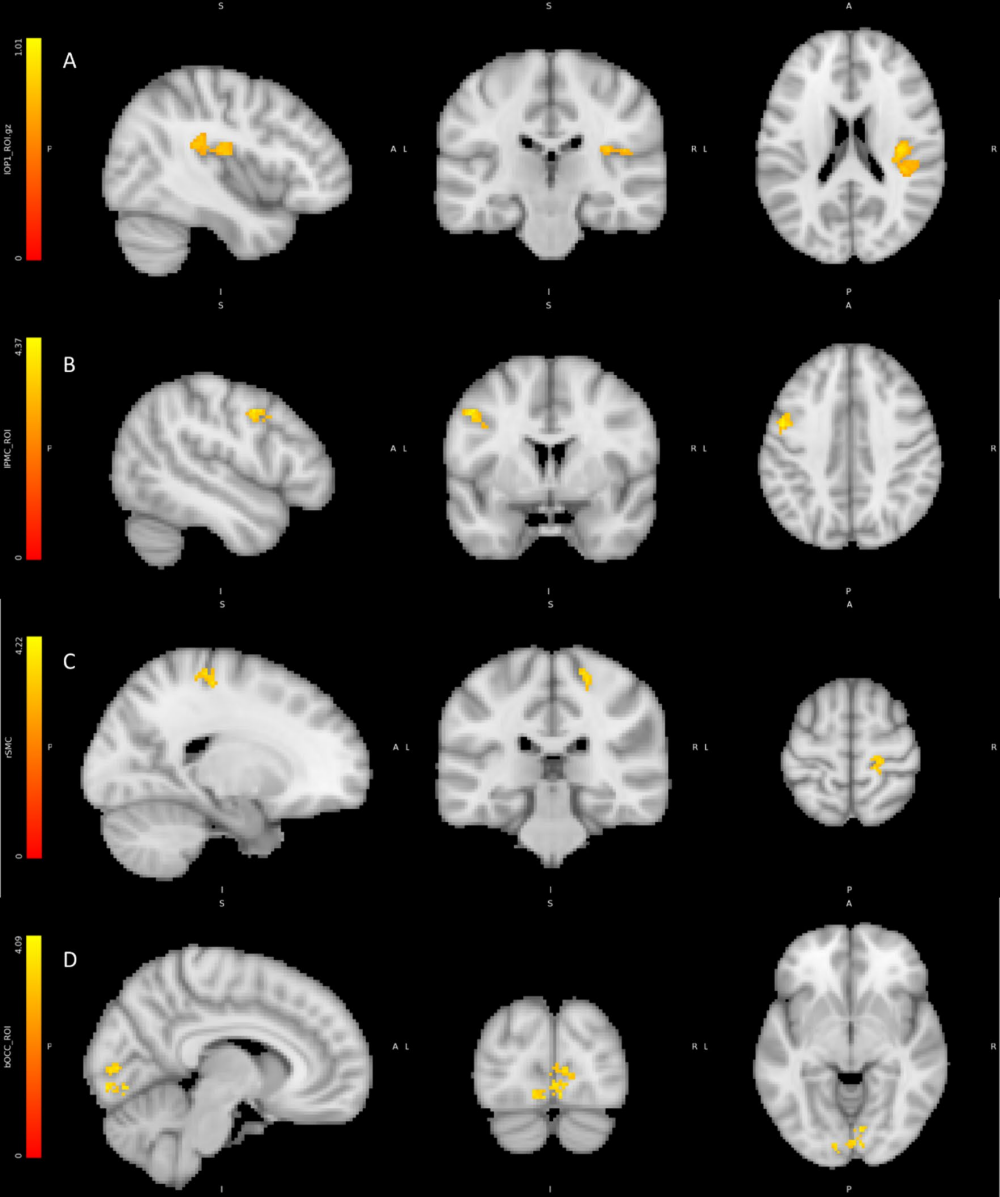
Regions correlating with any feedback vs. null contrast: (A) Right posterior insula and secondary somatosensory cortex. (B) Left premotor cortex. (C) Right sensorimotor cortex (D) Bilateral occipital cortex. Colour bars represent significant cluster-corrected z-statistics.

We tested for neural activity (differences in regional BOLD signal) to identify which regions of the brain were significantly activated by exposure to physiological feedback of any kind (Veridical & False) by contrasting trials in blocks containing feedback (HIGHER, LOWER, SAME) with blocks where there was no feedback (NULL), collapsing over valence using the Valence Model. This resulted in significant activations within the right posterior insular and secondary somatosensory cortex, ipsilateral to the side in which feedback was presented. Further regions including the left ventral premotor cortex, contralateral to the feedback mechanism and left sensory motor activation across Brodmann areas BA2 – BA6 ipsilateral to the feedback mechanism, indicative of a motor evoked potential (MEP) (See Table 3; Fig. 5).

**Table 3:** BOLD activity correlating with the contrast between all active feedback conditions (HIGHER, LOWER, SAME) vs. NULL feedback conditions.

Region	Hemisphere	Co-ordinates	Number voxels	t score
OP1/posterior insula	R	45.5–30.5 27.5	328	4.73
Primary visual cortex	L/R	−10.5 −88.5 −12.5	170	4.05
Premotor	L	−48.5 − 0.05 39.5	103	4.33
Sensory motor	R	21.5–26.5 57.5	83	4.18

**Fig. 6:**
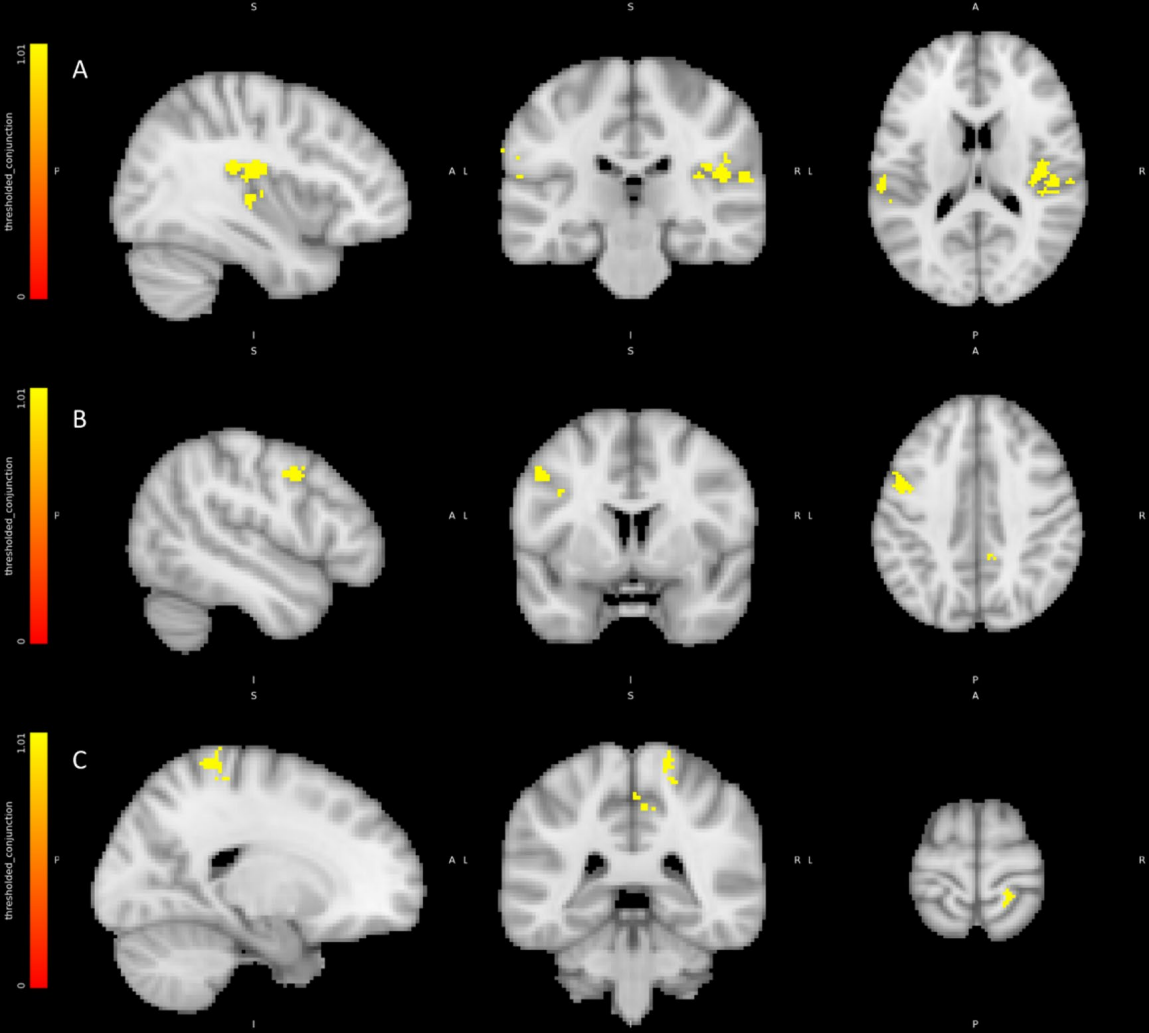
Conjunction analysis for feedback vs. null contrast corroborating initial analyses: (A) Right Posterior Insula and Bilateral somatosensory cortex (B) Left premotor cortex (C) Right sensory motor cortex. Colour bars represent significant cluster-corrected z-statistics.

For corroboration, we undertook a simpler analysis using the Feedback Model. Each level of feedback (HIGHER/LOWER/SAME) was independently contrasted against no feedback. A Bonferroni corrected conjunction analysis was then performed on the contrasts to reveal contralateral activations of the secondary somatosensory cortex (see Fig. 6) premotor cortex and ipsilateral posterior insula alongside regions present in the previous contrast.

#### Right posterior, middle and anterior insular activations predict the rate of false physiological feedback

**Fig. 7:**
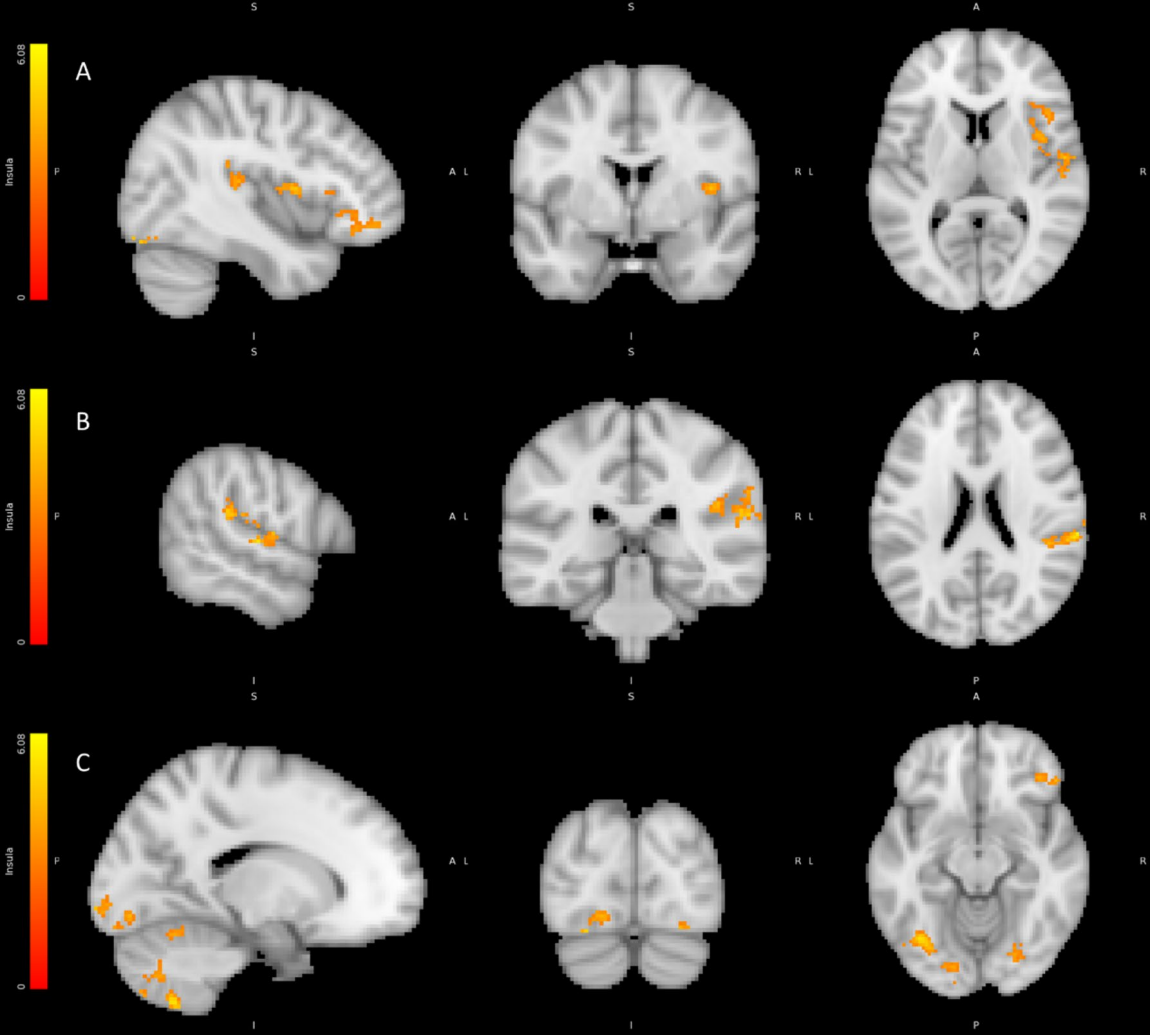
Regions correlating with HIGHER FFB vs. LOWER FFB contrast: (A) Right posterior middle and anterior insula and right orbitofrontal cortex. (B) Right supramarginal and superior temporal gyri and opercular cortex. (C) Bilateral fusiform cortex and cerebellum and right orbitofrontal cortex. Colour bars represent significant cluster-corrected z-statistics.

To test the hypothesis that activation in the right anterior insula (RAI) is associated with effects of FFB we contrasted trials during HIGHER FFB against trials during exposure to LOWER FFB using the Feedback Model. We found activations in both posterior and anterior regions of the right insula as well as activations in the right prefrontal cortex pars opercularis and pars orbitalis, right superior temporal gyrus, inferior supramarginal and bilateral occipital fusiform gyrus (see Table 4; Fig. 7). Thus, increases in false physiological feedback signals correlated with increased activity in right hemisphere cortical regions along a posterior-to-anterior axis encompassing regions implicated in primary viscerosensory representations (posterior insula) through to regions implicated in subsequent remapping and cross-modal integration of interoceptive information with exteroceptive sensation and motivational states^36^.

**Table 4:** BOLD activity correlating with the contrast between HIGHER FFB conditions vs. LOWER FFB conditions.

Region	Hemisphere	Co-ordinates	Number Voxels	t score
Cerebellum	R	17.5–60.5 −56.5	538	5.41
FFG/FFA	L	−36.5 −66.5 −12.5	250	5.5
Supramarginal/OP1/posterior insula	R	63.5–28.5 21.5	210	4.46
Occipital	L	−12.5 −96.5 −4.5	156	6.02
Middle/anterior insula	R	41.5 3.5 9.5	130	4.67
Cerebellum	L	−18.5 −60.5 −56.5	127	5.8
Orbitofrontal	R	49.5 33.5–14.5	125	5.23
FFG	R	41.5–78.5 −18.5	84	4.75
Superior temporal	R	57.5–16.5 5.5	70	5.4

#### Left secondary somatosensory cortex reflects the interaction between FFB and emotion

**Fig. 8:**
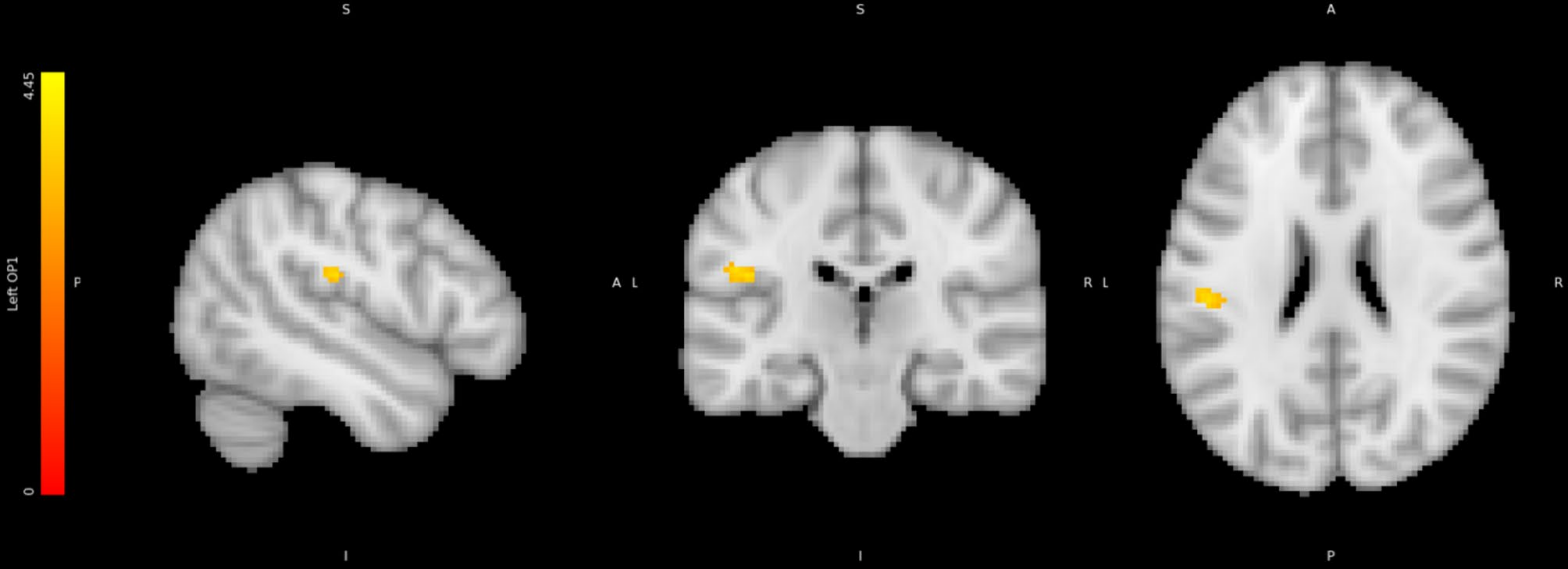
Region correlating with HIGHER FFB vs. LOWER FFB vs. positive emotions vs. negative emotions interaction contrast: Left Opercular Cortex. Colour bars represent significant cluster-corrected z-statistics.

To test the Effects of HIGHER vs. LOWER FFB on neural responses to emotional faces, we tested for neural activity reflecting the interaction of HIGHER/LOWER FFB*POSITIVE/NEGATIVE face valence using the Valence Model. Activations within the left secondary somatosensory/opercular cortex reflected this interaction. (See Table 5; Fig. 8).

**Table 5:** BOLD activity correlating with the interaction contrast between FFB (HIGHER/LOWER) * valence (POSITIVE/NEGATIVE).

Region	Hemisphere	Co-ordinates	Number voxels	t score
Secondary somatosensory/opercular cortex	L	−50.5 −20.5 21.5	52	4.41

#### Activity within primary somatosensory cortex predicts Time-dependent effects of FFB to negative emotions

**Fig. 9:**
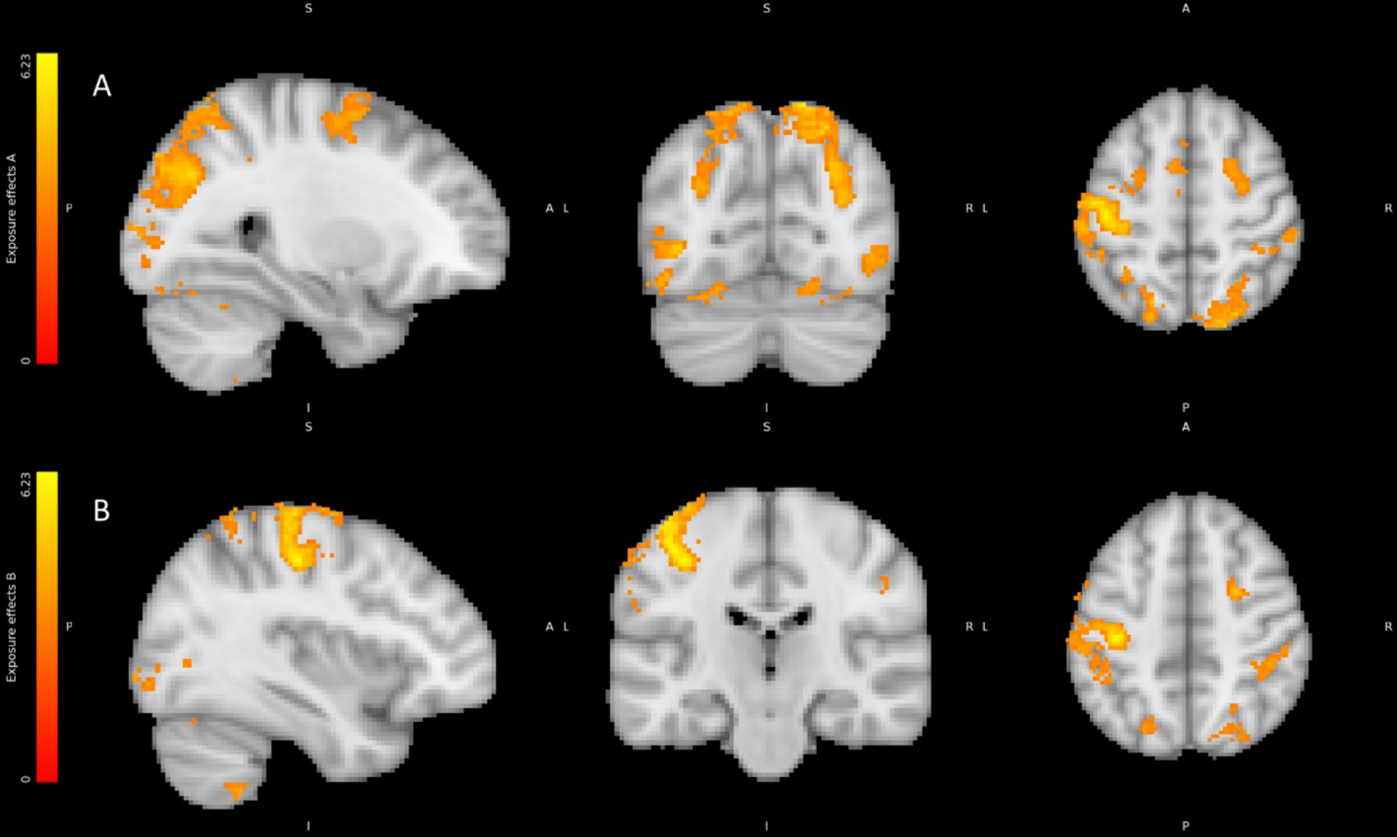
Regions correlating with HIGHER FFB vs. LOWER FFB during negative emotion trials. Colour bars represent significant cluster-corrected z-statistics.

Finally, to isolate neurophysiological substrates reflecting the core behavioural effects of FFB, we tested for neural correlates of exposure to FFB over time on processing of valenced face stimuli using the Exposure Model. We thus contrasted condition (HIGHER vs. LOWER) for positive and negative valence separately using the parametric modulation by trial number within stimulation blocks. No significant activation correlated with these contrasts during POSITIVE trials, therefore only NEGTATIVE contrasts are reported here. Suprathreshold activity across parietal, temporal, and frontal regions were specifically revealed by the contrast of HIGHER FFB against LOWER FFB only during Negative valence trials. Specifically, co-activity within right superior parietal and right intra-parietal sulcus, left central sulcus, right precentral gyrus, and sulcus, left supplementary motor cortex, and superior frontal sulci, attested to engagement of primary somatosensory representation with substrates supporting bodily centred representation in biasing emotional appraisal of face stimuli (see Table 6; Fig. 9). Correspondingly, lateral occipital sulci, and occipital and temporal fusiform cortices were concurrently engaged in this interaction.

**Table 6:** BOLD activity correlating with the interaction contrast between HIGHER FFB conditions vs. LOWER FFB conditions parametrically modulated by exposure in NEGATIVE Valence trials.

Region	Hemisphere	Co-ordinates	Number voxels	t score
Central sulcus	L	-40.5 -22.5 51.5	2507	6.17
Superior parietal sulcus	R	15.5–72.5 63.5	1735	5.79
Superior frontal sulcus	R	25.5 1.5 49.5	362	4.88
Inferior lateral occipital sulcus	R	43.5–76.5 -8.5	301	5.12
Inferior lateral occipital sulcus	L	-38.5 -72.5 1.5	281	5.25
Precentral sulcus	L	-56.5 7.5 41.5	280	4.51
Intra parietal sulcus	R	43.5–36.5 49.5	261	4.28
Cerebellum	R	17.5–54.5 -16.5	153	4.8
Supplementary motor cortex	L	-2.5 -2.5 61.5	148	4.29
Occipital fusiform gyrus	L	-20.5 -76.5 -18.5	117	4.22
Temporal occipital fusiform cortex	R	35.5–58.5 -22.5	110	4.14
Precentral gyrus	R	61.5 7.5 27.5	96	4.3
Cerebellum	R	11.5–62.5 -44.5	72	4.26
Occipital fusiform gyrus	R	17.5–70.5 -16.5	69	4.2
Cerebellum	L	-34.5 -50.5 -52.5	47	4.46
Occipital pole	L	-12.5 -92.5 -16.5	44	4.11

## Discussion

This study investigated emotion perception under the effects of false physiological feedback using pulsatile somatosensory stimulation of the wrist to emulate sensations of different heart rates. We examined neural and physiological mechanisms underpinning observed behavioural effects. Our initial analysis revealed no average effect of FFB, delivered over blocks of 20s on reported intensity ratings of emotional faces. However, a more granular examination revealed that the impact of FFB on these emotional ratings developed with exposure: Increased exposure, when stimulation was faster than veridical HR, amplified positive ratings of positive faces and negative rating of negative faces. Similarly, with increased exposure, when stimulation was slower than veridical HR, valence-congruent intensity ratings were attenuated. These behavioural effects were greatest for ratings of negative emotions. Analyses of neuroimaging data showed that heartbeat-like somatosensory stimulation reliably activated somatosensory and sensorimotor cortices including more viscero-sensory secondary somatosensory and posterior insula regions. Moreover, activity across a posterior-to-anterior swathe of right insular cortical regions differentiated FFB type (HIGHER/LOWER). This insular trajectory is implicated in interoceptive representation and integrative remapping of internal physiological signals, ultimately projecting into ventral and orbital prefrontal cortices implicated in social and emotional decision-making^19,23,36^. There was also concomitant recruitment of regions implicated in proprioceptive bodily (supramarginal gyrus) and visual object (fusiform gyrus) representations. Activity within left secondary somatosensory cortex reflected the interaction between emotional valence (POSITIVE/NEGATIVE) and FFB type (HIGHER/LOWER). Lastly, multiple areas, notably including primary somatosensory cortex, tracked the increasing impact of exposure over time to different rates of FFB (HIGHER/LOWER) on judgments of negatively valenced stimuli. Physiological data indicated no entrainment during the blocks of FFB.

Our study extends published research in the field of FFB: We built upon the observation that ambiguous emotional judgements (neutral faces) were most influenced by FFB^11^ and presented emotional faces on a continuum of emotionality that was biased towards neutral faces to increase reliance on interoceptive processes while maintaining emotional valence^11^. Importantly, our participants were exposed to *somatosensory vibro-tactile* stimulation, rather than auditory feedback.

When we looked at the impact of FFB on emotional ratings, our initial analyses of the average effect of the feedback blocks found no main effect of Condition (faster or slower simulated heart rate), nor interaction between Condition and Emotion. This contrasted with earlier findings using auditory^9,11^ and even somatosensory stimulation^26,37^. Aspects of our pseudorandomised task design may be relevant: e.g. there was no intervening rest period between different FFB stimulation blocks, hence ‘leakage effects’ of the previous condition (and potentially transient psychophysiological orientating responses to the switch in stimulation rate) may have affected the first few trials in the next FFB block. This leakage effect from the preceding condition is suggested by our data (see Fig. 3), wherein Trial one of LOWER blocks has on average a greater intensity rating and Trial one at HIGHER FFB starts at an intensity rating below neutral.

However, when we accounted for evolving effects of false physiological feedback over time, this improved the specificity of the model to reveal a robust bi-directional effect of FFB type on subjective intensity ratings. This bi-directional effect was more complex than effects reported in prior investigations using auditory FFB. For example, auditory FFB ascending or descending in rate both increased attractiveness ratings of photos^9^. Similarly, the main effect of pulsatile auditory FFB (both faster and slower than veridical HR) increased intensity ratings of neutral faces^11^. Nevertheless, our findings with somatosensory FFB mirror the reported reduction in anxiety during slow somatosensory FFB^26^, suggesting there is a qualitative difference between somatosensory and auditory FFB in how changes impact emotional processing. Importantly we found no entrainment of heart rate to different rates of somatosensory stimulation, consistent with the notion that interoceptive/autonomic mismatch^11,13^ rather than physiological reactivity drives the behavioural effects of FFB.

Brain regions engaged when processing pulse-like somatosensory stimulation to the wrist included substrates for both somatosensory and interoceptive processing. However, the subthreshold activation of contralateral primary somatosensory cortex (S1) reflects the low magnitude, infrequent and transient nature of this pulse sensation, possibly relayed by (unmyelinated) c-tactile fibres. The fMRI sensitivity to activation within regions that run perpendicular to the skull – including the wrist representations within primary somatosensory cortex – is relatively low^30^. Nevertheless, in blocks where stimulation was delivered, we observed engagement of bilateral secondary somatosensory cortices (S2) alongside right sensorimotor, right posterior insula, and left ventral premotor cortex. Here, bilateral S2 activation to unilateral somatosensory stimulation^38^ suggests the conversion of a sensation into a perception, beyond the direct representation of the sensation itself^39^. Moreover, the light pulsatile stimulation at the wrist, delivered at an intensity on the border of conscious awareness and spatially distributed, is unlikely to have activated the deeper specialised somatosensory mechanoreceptors that support discriminatory touch and its representation within S1. Instead, our FFB, with its neural representation within S2, insula and bilateral parietal opercular regions, and its impact on emotional processes, are more akin to what is observed for affective touch evoked by repeated light brushing of the skin^40–43^. Interestingly, light repetitive stimulation of the skin, activating unmyelinated c-tactile fibres, can engender ambivalent sensations such as tickle and itch. These may indicate infestation and evoke the initiation of ‘defensive’ reactions or behaviours^44^.

Correspondingly, we observed the engagement of contralateral (left) premotor cortex, a region implicated in the discriminatory categorisation of somatosensory sensations as self(body)-owned or as an external threat to personal space. Neurons in primate ventral premotor cortex (F4) map peri-personal space^45^ and may initiate defensive motor reactions to visuo-tactile threats^46,47^. Thus, the region integrates multimodal sensory information within representations of peri-personal space and bodily action^48^. In humans, ventral premotor cortex, is engaged by illusions of body ownership (rubber hand illusion)^49,50^ and tracks the multisensory perceptual representation of the whole-body^51^. In our own study, the engagement of ventral premotor cortex by somatosensory sensory feedback likely indicates representational tuning of threat/non-threat decisions regarding the origin and ownership of the touch sensation, a process that would not be relevant to auditory (or visual) FFB paradigms. By extension, activation of premotor cortex by FFB signals deemed to be self-owned (i.e. internally generated/ interoceptive) may nevertheless predispose to reflexive movement and thus recruit motor inhibitory pathways, including those connecting contralateral premotor and ipsilateral motor cortices^52–54^.

When we examined where in the brain a simulated state of higher cardiovascular arousal was represented during emotional judgement of faces, we observed the engagement of regions implicated in a caudo-rostral stream of bodily and interoceptive representation and its integration, specifically in the inferior supramarginal gyrus, right dorsal posterior, middle and anterior insula, through to ventrolateral and orbital prefrontal cortex. Increasing simulated arousal also enhanced activity within regions implicated in processing of visual information, including emotional expressions of others, namely right superior temporal gyrus, bilateral occipital fusiform gyrus.

Posterior insular cortex supports a primary mapping of visceral bodily signals, however here the sensory stimulus is somatosensory/vibrotactile. Our observation is nevertheless supportive of Craig’s proposed inclusion of affective touch (via tactile c fibres) within (Laminar 1 spinothalamic) within interoception^55,56^. Affective touch modulates body ownership in rubber hand illusion paradigms^57,58^. Not only is posterior insula activated by changes in autonomic arousal, but it is also brought online by attentional awareness and interpretation of physiological states^21,55,59,60^. Sensory-motor afferents reaching this region^61^, may permit a somatosensory contribution to the cortical representation of physiological signals and the interpretation of their affective meaning^56,60^.

The projection of interoceptive information through insula along a posterior-anterior gradient is proposed to support contextual integration at increasing levels of complexity^23,36,55^, for example in the thermal grill illusion where primary representation of temperature in posterior insula is accompanied by the integrated, subjective conscious experience of temperature within anterior insula^62^. Mid insula lies somewhere between, potentially supporting some subjective bodily experiences^55^, where interoceptive information can underpin the representation of the ‘biological self’^63^ including the sense of body ownership engendered by the rubber hand illusion^64^.

Increased right anterior insula (AIC) activity was observed to correlate with asynchronous over synchronous heartbeats in a previous (auditory) false physiological feedback paradigm^11^. This region is implicated in the integration of interoceptive and exteroceptive signals, and sensitive to prediction errors^18,20–22,55,59^. In this context representations within anterior insula appear to be accessible to conscious awareness, giving rise to the experience of visceral, motivational, and emotional states^21,22^. In risky decision-making, activity here reflects uncertainty^65^ and correlates with feelings of anticipated value^66^. Changes in the perceived emotional intensity of neutral faces is reported to evoke anterior insular activity via a mismatch between exteroceptive feedback and veridical interoceptive signalling^11^. In the present study, bi-directional, feedback dependent, changes in emotional intensity ratings that suggest AIC activity is playing a more complex role that is consistent with somatosensory feedback being a more ‘interoceptively valid’ signal for conscious appraisal that can contribute predictively to mechanisms through which the biological self interacts with the representation of salient emotional features in the external environment^67^.

Anatomically, anterior insula projects into ventrolateral and orbital prefrontal cortical regions implicated in value-driven behavioural flexibility: These regions were engaged by simulated arousal during emotional face processing and are known to respond to faces^68–70^ and the perception of other’s emotions^71,72^. Correspondingly, these areas contribute to emotional decision-making^73–76^ and their dysfunction impairs face recognition^77–79^ and social communication^80^.

We have demonstrated that arousal-like false physiological feedback enhances activity within two other sensory cortical regions involved in face processing, the superior temporal gyrus and fusiform cortex, attesting to the transfer of embodied ‘arousal’ to perceptual salience^81,82^. Typically, negative valence faces carry greater behavioural salience, and superior temporal gyrus is likely involved in the top-down representation of such salience^11,83,84^, and its exaggeration in clinical depression^85^. Face-responses within fusiform cortex, while more sensitive to identity than emotion^86–90^ are also enhanced by salience and attention, encoding the level of psychophysiological arousal alongside amygdala, a region involved in threat detection^11,21,91^.

We observed that integration of simulated interoceptive information concerning arousal with the valence information from each face was associated with activity changes within the left parietal opercular region which extends to secondary somatosensory cortex. This region integrates representations across sensory modalities, particularly visual and somatic^92,93^. S2 also supports working memory within the somatosensory/tactile domain^94^ perhaps underpinning the observed evolving effect of FFB exposure. Interestingly, activity here correlates with the integration of prior information to bias the perceptual encoding of novel stimuli in the context of a vibro-tactile magnitude discrimination test^95^. One functional mechanism suggested by these authors is the local instantiation of Bayesian inference within probabilistic neuronal population codes^96–98^. Considering how this interpretation can be applied to the current investigation, an uncertain representation of face valence (biased towards neutral) and subsequent perceptual decision about its intensity, is combined with the cumulative representation of interoceptive signals for the FFB block, biasing emotional perception of the stimuli.

Behaviourally, the most striking finding from the present study was the time dependent build-up of effects of false physiological feedback on emotional ratings. This shows accumulation of evidence, over the course of each 20s stimulation block, about putative arousal state (or unattributed arousal) implied by the pulse-like sensation. Our neuroimaging investigation of the neural correlates of this time-dependent effect on perceptual appraisal of the faces, revealed effects that were significant for negative faces, but not positive faces. Within the brain, activity ramped up across distributed regions encompassing parietal, primary sensorimotor and supplementary motor cortex regions, cerebellum, fusiform and lateral occipital cortices, and superior frontal cortex. This pattern attests to the incremental engagement of cortical centres in the assimilation of a bodily signal (albeit false feedback physiological state) into the processing of emotional salience. Speculatively, this suggests that prediction error within cross modal representations of bodily state is actively resolved through the affective weighting of foreground visual face information in an attention-dependent manner. This mirrored what we observed in the behavioural interaction where effect size was greater for negative, compared to positive, face processing. The small effect during positive trials was likely unable to survive correction during the neuroimaging analysis which does not necessarily preclude its existence but invites future investigation into the exposure effects of FFB on positive emotion appraisal.

One point of importance as it relates to the interpretation of the neural data is the laterality of feedback stimulation, as we subjected all participants to stimulation on the right wrist. We found increased activity in the insula of the right hemisphere during feedback of any type and when the frequency of feedback signals was increased. This increase in ipsilateral excitation not just in the insula but across several socioemotional regions elicited when increasing the frequency of feedback signals is unexpected when viewed purely from the perspective of contralateral organisation. However, in line with the work from Craig^19,36,55,59^, finding hemispheric lateralisation on the right (ipsilateral) hemisphere as a correlate of external feedback bolsters the claims made in this paper that somatosensory stimulation at the wrist is eliciting an interoceptive response, which in turn, effects changes in emotion perception and decision-making. It is likely that other, non-lateralised processes in the brain, such as threat detection and integration of body ownership are processed contralateral to the site of stimulation. Findings by Preuschof and colleagues (2009) isolated prior information biases within a predictive coding model contralateral to the site of stimulation, similar to the present investigation, however, both studies presented stimulation on the right side of the body, preventing any investigation of laterality for this process. An identical paradigm in which the site of stimulation was flipped would improve the literature on lateralisation of interoceptive processes, predictive coding and the nuance of somatosensory processing in the brain.

In summary, pulsatile somatosensory simulation, emulating the heart beating at different heart rates, engendered an incremental biasing of the perceived emotionality of face images. Within the brain, such stimulation engaged neural regions in ways that indicated an initial decision process toward embodiment, enhancing activity within cortical areas tuned to body ownership and the detection of personal threat. Recruitment of regions including secondary somatosensory cortex suggested processing similar to affective touch, ultimately relaying to anterior insular and ventral prefrontal cortices. Here, this interoceptive representation of pulsatile somatosensory stimulation diverges from effects of auditory false physiological feedback: The processing of the somatosensory false physiological feedback is tracked through the insula’s posterior to anterior axis of interoception into ventral prefrontal regions that support emotional decisions. Nevertheless, progressive exposure to the false feedback signals in the context of negative appraisals, shifted activity across a network of neocortical regions that support the integrated representation of bodily position, action generation and visual information, within the immediate sensorium. Together, these findings extend our understanding of the effects of FFB on emotional experience and behaviour, highlighting predictive bodily mechanisms in the attribution of salience.

However, limitations include the block design of the FFB paradigm, in which there was no (jittered or fixed) period of absent stimulation between each 20 s block likely engendering leakage of effects between blocks (as shown in Fig. 3) that may have reduced discriminatory power with potential implications for neuroimaging analyses. Moreover, the initial findings of Valins (1966) have raised concerns about demand characteristics^99^, wherein participants’ responses to ‘perceived’ change in heart rate may reflect the effect seemingly desired by the experimenters. However, we presented the somatosensory feedback at a weak (peri-liminal) intensity that was individually tailored for each participant, such that when debriefed after the experiment, most participants reported little or no awareness of the somatosensory sensation when their attention was focussed on the face paradigm, mitigating effects of demand characteristics. However, this process also introduced subjective variability. Other considerations include the predominantly female participant sample, and our behavioural testing within an MRI environment, in contrast to studies in real world scenarios^26,100^. We included two negative emotion types to ensure inferences about perceived negative emotion were generalizable while leaving open the possibility of further analyses to differentiate fear from anger as seen in earlier work^101^. This could be the driver behind the interaction between FFB and negative emotions in both behavioural and neural data, as participants were much less likely to habituate to negative emotions due to its variability of expression. Additionally, four individual identities were used to ensure that each intensity value per valence was represented by the same faces, best efforts to standardise this were made by using two male and two female face sets, but the lack of variability hinders the ecological validity of the data. The feedback conditions are constrained by not modelling a stochastic rhythm capable of mimicking the natural variability in IBI’s of veridical heart rates. As such, the behavioural effects of these conditions and subsequent analysis of the neural data should be interpreted with this in mind.

The cognitive drivers and neural pathways utilised by somatosensory FFB as a mediator of emotional bias have received little attention until now. One aspect highlighted by this investigation is the potential for distinct modalities of false physiological feedback to mimic physiological arousal states and be interpreted interoceptively (hence emotionally) to differing degrees. Future research is needed to unpack this empirically and/or combine modalities (somatosensory/auditory/visual) to maximise the perceived veracity and impact of false physiological feedback, for example using virtual reality^102–104^.

In conclusion, this study provides behavioural evidence for a bi-directional effect of somatosensory FFB on intensity ratings of emotionally ambiguous faces over time, with physiological evidence contributing to the accepted narrative that effects of FFB are driven by cognitive appraisal mechanisms rather than physiological entrainment. Our observations provide evidence that pulsatile somatosensory sensation may be integrated into a bodily owned representation potentially via pathways linked to affective touch. This embodiment, and its appraisal as self-generated autonomic information, impacts the emotional processing of faces. Our findings appear qualitatively different to research using sound as a false feedback signal. Thus, the modality of stimulation, its intensity and exposure time are all important contributing factors for consideration in future research into somatic contributions to the emotional experience.

## Electronic supplementary material

Below is the link to the electronic supplementary material.

Supplementary Material 1

## Data Availability

The raw behavioural and pre-processed physiological datasets generated during and/or analysed during the current study are available in the OSF repository, https://osf.io/xjah4/. The neuroimaging datasets generated during and/or analysed during the current study are not publicly available due to file size restrictions but are available from the corresponding author on reasonable request.
