# Supplementary material for: Somatosensory false feedback biases emotional ratings through interoceptive embodiment
Source: Sci Rep. 2025 Apr 3;15:11472. doi: 10.1038/s41598-025-94971-6 (PMC11968835; doi:10.1038/s41598-025-94971-6)
Supplement: Supplementary file 1 — Supplementary Material 1 [file 41598_2025_94971_MOESM1_ESM.pdf]

Title: Somatosensory false feedback biases emotional ratings through interoceptive embodiment

Authors: Patchitt, J.,[1,2] Garfinkel, S.,[3] Strawson, WH.,[2] Miller, M.,[4,5] Tsakiris, M.,[6] Clarke A.,[7,8] Critchley, HD.[1,2,8]

Affiliations:

1 Sussex Centre for Consciousness Science, University of Sussex, Brighton, BN1 9QJ, UK.

2 Department of Clinical Neuroscience, Brighton and Sussex Medical School, University of Sussex, Brighton, BN1 9RY, UK.

3 Institute of Cognitive Neuroscience, University College London, London, WC1N 3AZ, UK.

4 Monash Centre for Consciousness and Contemplative Studies, Monash University, Melbourne, VIC 3168, Australia.

5 Psychology Department, University of Toronto, Toronto, ON M5S 2E5, Canada.

6 Department of Psychology, Royal Holloway, University of London, Egham, TW20 0EX, UK

7 School of Media, Arts and Humanities, University of Sussex, Brighton, BN1 9RG, UK.

8 School of Engineering and Informatics, University of Sussex, Brighton, BN1 9QG, UK.

Corresponding authors: Joel Patchitt (j.patchitt@bsms.ac.uk)

Inclusion/Exclusion criteria

Participants were self-declared free from illness, disability, and psychoactive medication with normal or corrected-to-normal vision (i.e. glasses, contact lenses, laser eye surgery). No excessive consumption of alcohol (<10 glasses per week), caffeine (equivalent of 2 cups of regular coffee per day) or any illegal drugs two months prior to the study. Additional criteria for the fMRI session included no irreversibly attached metal in the body, claustrophobia, dermal plates, or contraceptive coils. Participants were recruited using convenience sampling methods within a short timeframe, with the maximum sample size being limited by a tight funding deadline.

Emotional Face Stimuli

Eckman faces of each emotion type were biased so that out of the 10 images used, 4 were completely emotionally ambiguous (0, 1, 2, 4; as labelled in the Eckman database), 3 were very ambiguous (6, 8, 10) and 3 ranged from distinguishable to obvious (12, 16, 20) (see Fig. A).

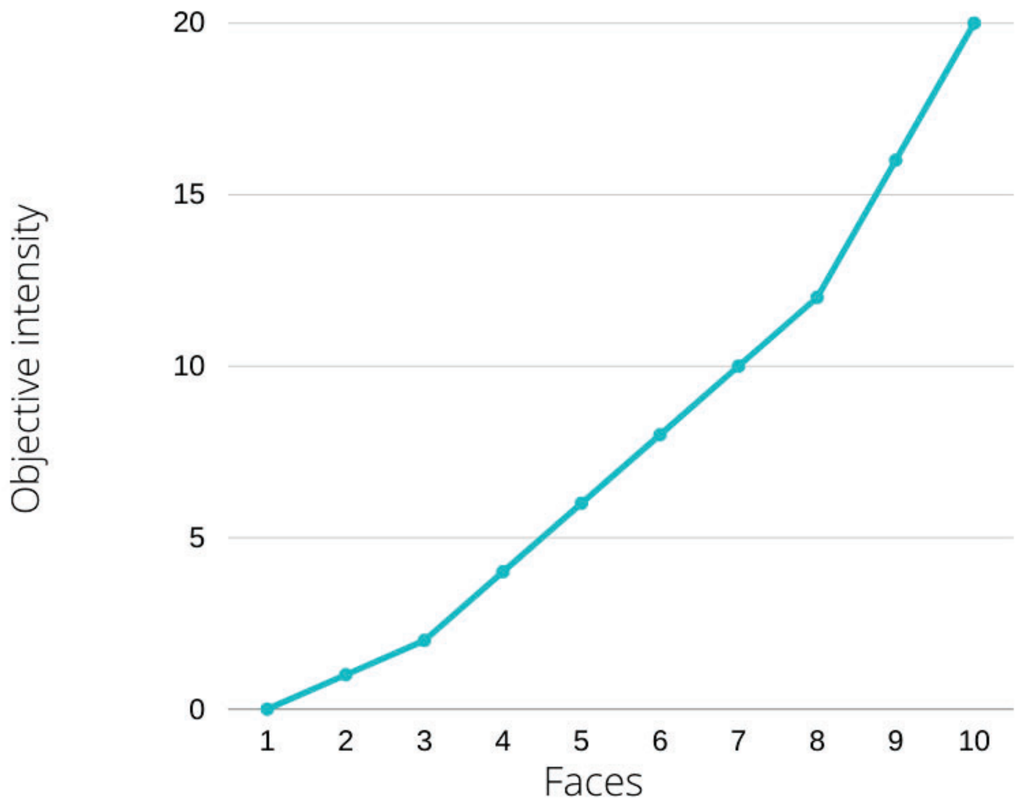

Fig A. Visualisation of the image selection with objective intensity of the face as labelled by the Eckman file system on the y axis totalling 10 images as depicted on the x axis.

fMRI BOLD Scanning Parameters

**Manufacturer**	Siemens
**Field Strength**	3T
**Model Name**	Prisma
**No. of sessions**	1
**Volumes acquired**	899

**Pulse Sequence Type**	Spin Echo, EPI
**Field of View**	208x208mm
**Matrix Size**	97x115x97
**Slice Thickness**	2mm
**No. of Slices**	72
**Acquisition Orientation**	Axial
**Whole Brain**	Yes
**Slice Acquisition Order**	Interleaved
**TE**	28ms
**TR**	1520ms
**Multi-band Factor**	3
**Flip Angle**	75 degrees

Pre-processing of B_0 Inhomogeneity Mappings

A B_0-nonuniformity map (or *fieldmap*) was estimated based on two (or more) echo-planar imaging (EPI) references with topup (Andersson, Skare, and Ashburner (2003); FSL 6.0.5.1:57b01774).

Anatomical Data Pre-processing

The T1-weighted (T1w) image was corrected for intensity non-uniformity (INU) with N4BiasFieldCorrection (Tustison et al. 2010), distributed with ANTs 2.3.3 (Avants et al. 2008, RRID:SCR_004757), and used as T1w-reference throughout the workflow. The T1w-reference was then skull-stripped with a *Nipype* implementation of the antsBrainExtraction.sh workflow (from ANTs), using OASIS30ANTs as target template. Brain tissue segmentation of cerebrospinal fluid (CSF), white-matter (WM) and grey-matter (GM) was performed on the brain-extracted T1w using fast (FSL 6.0.5.1:57b01774, RRID:SCR_002823, Zhang, Brady, and Smith 2001). Brain surfaces were reconstructed using recon-all (FreeSurfer 6.0.1, RRID:SCR_001847, Dale, Fischl, and Sereno 1999), and the brain mask estimated previously was refined with a custom variation of the method to reconcile ANTs-derived and FreeSurfer-derived segmentations of the cortical grey-matter of Mindboggle (RRID:SCR_002438, Klein et al. 2017). Volume-based spatial normalization to one standard space (MNI152NLin2009cAsym) was performed through nonlinear registration

with antsRegistration (ANTs 2.3.3), using brain-extracted versions of both T1w reference and the T1w template. The following template was selected for spatial normalization: *ICBM 152 Nonlinear Asymmetrical template version 2009c* [Fonov et al. (2009), RRID:SCR_008796; TemplateFlow ID: MNI152NLin2009cAsym].

Functional Data Pre-processing

For each of the 3 BOLD runs found per subject (across all tasks and sessions), the following pre-processing was performed. First, a reference volume and its skull-stripped version were generated by aligning and averaging 1 single-band references (SBRefs). Head-motion parameters with respect to the BOLD reference (transformation matrices, and six corresponding rotation and translation parameters) are estimated before any spatiotemporal filtering using mcflirt (FSL 6.0.5.1:57b01774, Jenkinson et al. 2002). The estimated *fieldmap* was then aligned with rigid-registration to the target EPI (echo-planar imaging) reference run. The field coefficients were mapped on to the reference EPI using the transform. BOLD runs were slice-time corrected to 0.719s (0.5 of slice acquisition range 0s-1.44s) using 3dTshift from AFNI (Cox and Hyde 1997, RRID:SCR_005927). The BOLD reference was then co-registered to the T1w reference using bbregister (FreeSurfer) which implements boundary-based registration (Greve and Fischl 2009). Co-registration was configured with six degrees of freedom. First, a reference volume and its skull-stripped version were generated using a custom methodology of *fMRIPrep*. Several confounding time-series were calculated based on the *pre-processed BOLD*: framewise displacement (FD), DVARS and three region-wise global signals. FD was computed using two formulations following Power (absolute sum of relative motions, Power et al. (2014)) and Jenkinson (relative root mean square displacement between affines, Jenkinson et al. (2002)). FD and DVARS are calculated for each functional run, both using their implementations in *Nipype* (following the definitions by Power et al. 2014). The three global signals are extracted within the CSF, the WM, and the whole-brain masks. Additionally, a set of physiological regressors were extracted to allow for component-based noise correction (*CompCor*, Behzadi et al. 2007). Principal components are estimated after high-pass filtering the *pre-processed BOLD* time-series (using a discrete cosine filter with 128s cut-off) for the two *CompCor* variants: temporal (tCompCor) and anatomical (aCompCor). tCompCor components are then calculated from the top 2% variable voxels within the brain mask. For aCompCor, three probabilistic masks (CSF, WM and combined CSF+WM) are generated in anatomical space. The implementation differs from that of Behzadi et al. in that instead of eroding the masks by 2 pixels on BOLD space, the aCompCor masks are subtracted a mask of pixels that likely contain a volume fraction of GM. This mask is obtained by dilating a GM mask extracted from the FreeSurfer's *aseg* segmentation, and it ensures components are not extracted from voxels containing a minimal fraction of GM. Finally, these masks are resampled into BOLD space and binarized by thresholding at 0.99 (as in the original implementation). Components are also calculated separately within the WM and CSF masks. For each CompCor decomposition, the *k* components with the largest

singular values are retained, such that the retained components' time series are sufficient to explain 50 percent of variance across the nuisance mask (CSF, WM, combined, or temporal). The remaining components are dropped from consideration. The head-motion estimates calculated in the correction step were also placed within the corresponding confounds file. The confound time series derived from head motion estimates and global signals were expanded with the inclusion of temporal derivatives and quadratic terms for each (Satterthwaite et al. 2013). Frames that exceeded a threshold of 0.5 mm FD or 1.5 standardised DVARS were annotated as motion outliers. The BOLD time-series were resampled into standard space, generating a *pre-processed BOLD run in MNI152NLin2009cAsym space*. First, a reference volume and its skull-stripped version were generated using a custom methodology of *fMRIPrep*. All resamplings can be performed with *a single interpolation step* by composing all the pertinent transformations (i.e. head-motion transform matrices, susceptibility distortion correction when available, and co-registrations to anatomical and output spaces). Gridded (volumetric) resamplings were performed using antsApplyTransforms (ANTs), configured with Lanczos interpolation to minimize the smoothing effects of other kernels (Lanczos 1964). Non-gridded (surface) resamplings were performed using mri_vol2surf (FreeSurfer).

Post fMRIPrep Pre-processing

The following pre-processing steps were conducted outside of fMRI prep. The fMRIprep functional output was skull stripped via fslmaths by multiplying each voxel in the time-series by an fMRIprep generated *MNI152NLin2009cAsym space,* motion corrected, whole brain mask**,** multiplying any voxel outside the brain mask by zero. Functional scans were subsequently smoothed with a 6 mm Gaussian smoothing kernel, using a fully 3-dimensional plane and a local median filter in the event of single-point noise detection, after which the smoothed images were skull stripped once more using the fMRIprep generated *MNI152NLin2009cAsym space* whole brain mask. The first 5 functional volumes were discarded to allow for equilibration of net magnetisation and the final functional volumes collected were standardised across participants to 3 whole volumes after task end (bar one participant whose functional data contained only two full volumes after task end).

First Level Motion Regressor Selection

The *CompCcor* confound selection strategy using Nilearn's *load_confound_strategy* was implemented in selecting the first 6 *CompCor* motion regressors and fMRIprep's discreet cosine-basis high pass filtering regressors. These regressors joined the 6 head-motion estimates, their derivatives, and the powers of each resulting in 50 covariates of no interest being added to the model**.** The Autocorrelation function was estimated locally at each voxel, tapered, and regularized in space.

Spread of datapoints within the behavioural data

Datapoints per trial: 2650 each

Datapoints per condition: 3200 each

Datapoints per Emotion: 6499 each

*Datapoints per Condition*emotion combination: 1600 each*

*Datapoints per Condition*trial combination: 640 each*

*Datapoints per Emotion*trial combination:* ***Between 960-1440***

*Datapoints per Condition*Emotion*Trial combination:* ***Between 200-440***

The latter two figure ranges being due to the randomised presentation of face stimuli in blocks with an odd number of trials.

3 way behavioural interaction with SAME condition included

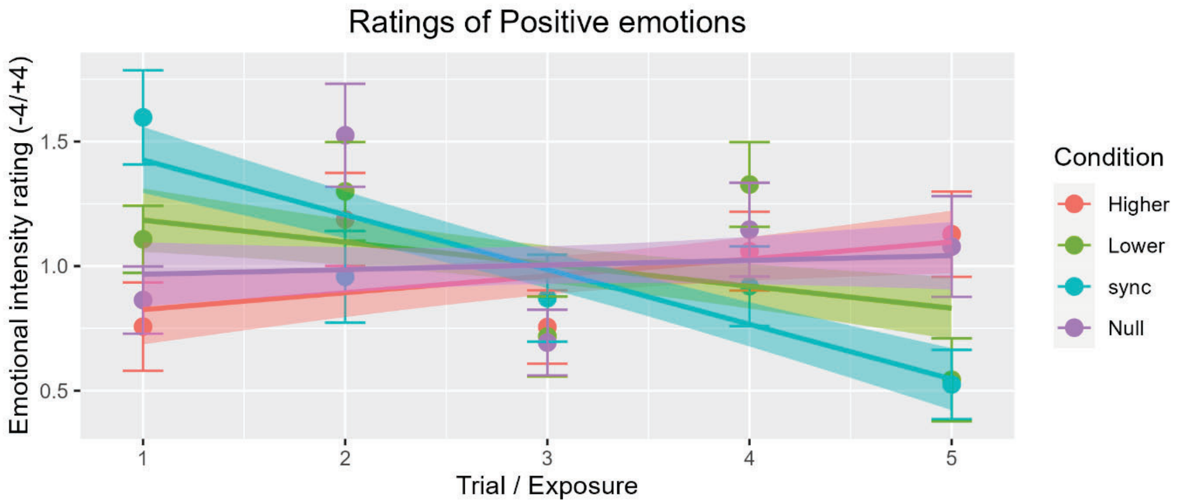

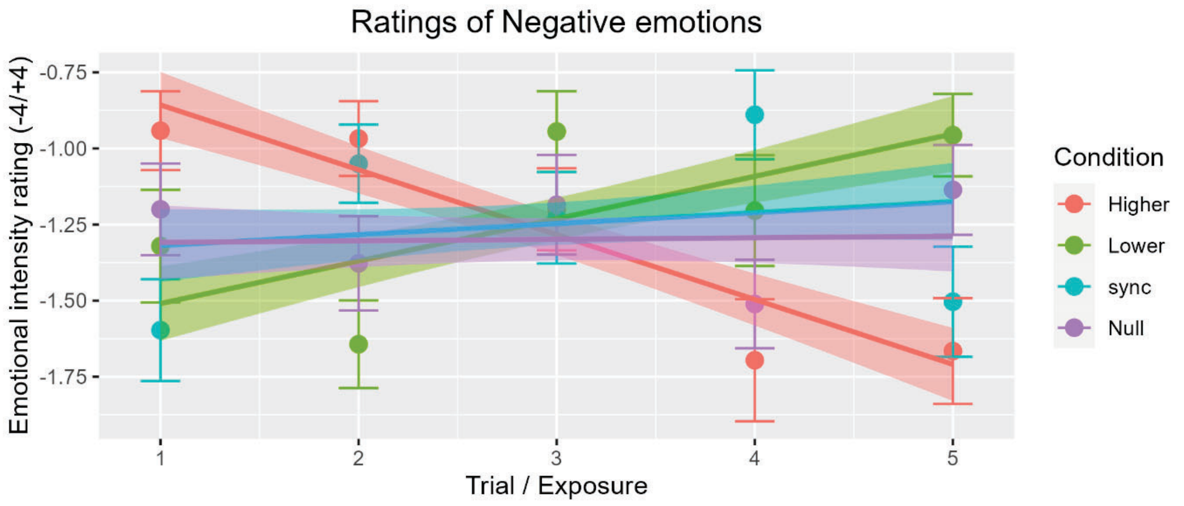

<u>**Alternative analysis of the behavioural data**</u>

We further characterised time-dependent FFB effects and contrasted effects of exposure against no FFB in the rating of positively and negatively valanced stimuli, by quantifying effect magnitude (least-squares means) for every combination of Condition and Emotion on the Trial factor within the linear mixed effects model and running (Tukey corrected) pairwise comparisons. We observed that ratings of POSITIVE faces showed time-dependent effects of LOWER FFB only, while NEGATIVE emotional faces showed time-dependent effects of both HIGHER and LOWER FFB (i.e. significant differences were revealed between exposure effects of LOWER FFB vs NULL FFB for both POSITIVE and NEGATIVE stimuli, and significant differences between the exposure effects of HIGHER FFB vs NULL FFB for judgements of NEGATIVE stimuli, but not POSITIVE stimuli).

Table 3. Tukey corrected pairwise comparisons conducted on the least-squares means for combinations of Condition and Emotion based on the Trial factor within the linear mixed-effects model				
	β^{Diff}	SE^{Diff}	***t(39)***	***p***
Higher Positive vs Null Positive	0.660	0.00004	1.337	.763

Higher Negative vs Null Negative	-0.218	0	-6.195	<.001
Lower Positive vs Null Positive	-0.275	-0.002	-3.013	.048
Lower Negative vs Null Negative	0.134	0.002	3.687	.008
